# Activity-dependent refinement of axonal projections forms one-to-one connection pattern in the developing chick ciliary ganglion

**DOI:** 10.3389/fncel.2025.1560402

**Published:** 2025-04-09

**Authors:** Ryo Egawa, Hiromu Yawo, Hiroshi Kuba

**Affiliations:** ^1^Department of Cell Physiology, Graduate School of Medicine, Nagoya University, Nagoya, Japan; ^2^Department of Developmental Biology and Neuroscience, Tohoku University Graduate School of Life Sciences, Sendai, Japan

**Keywords:** calyx synapse, synaptic competition, axon pruning, single-axon tracing, Brainbow labeling, tissue clearing, *in ovo* electroporation

## Abstract

Although it is well established that initially overproduced synaptic connections are extensively remodeled through activity-dependent competition for postsynaptic innervation, the mechanisms determining the final number of postsynaptic targets per axon remain unclear. Here, we investigated the morphology of individual axonal projections during development and the influence of neural activity in the chick ciliary ganglion (CG), a traditional model system for synapse maturation. By single-axon tracing combining Brainbow labeling and tissue clearing, we revealed that by embryonic day 14 (E14), hundreds of preganglionic axons each establish a one-to-one synaptic connection with single CG neurons via a calyx-type presynaptic terminal enveloping the soma of its postsynaptic target. This homogeneous connection pattern emerged through presynaptic terminal maturation from bouton-like to calyx-like morphology and concurrent axonal branch pruning starting around E10. The calyx maturation was retarded by the presynaptic expression of genetically encoded tools for silencing neuronal activity, enhanced tetanus neurotoxin light chain (eTeNT) or Kir2.1, demonstrating the activity-dependence of this morphological refinement. These findings suggest that some presynaptic mechanisms as well as synaptic competition would operate to restrict the number of postsynaptic targets innervated by each axon in the CG. Together with the easy accessibility to single-axon tracing, our results highlight the potential of the chick CG as a model for investigating the presynaptic factors underlying circuit remodeling.

## Introduction

During development, both the central and peripheral nervous systems undergo a refinement process involving the elimination of transiently overproduced synapses (Okawa et al., 2014; Faust et al., 2021). In various circuits, including the cerebellar climbing fiber-Purkinje cell synapse (Hashimoto et al., 2011; Kano et al., 2018; Kano and Watanabe, 2019) and the neuromuscular junction (Walsh and Lichtman, 2003; Buffelli et al., 2003), presynaptic axons compete for innervation of the same postsynaptic target in an activity-dependent manner, often leading to a “winner-takes-all” outcome where a single presynaptic axon establishes a stable synaptic connection while others are eliminated. Concurrently with this synaptic competition, individual presynaptic axons retract their exuberant branches with synapses, converging their connections onto a certain number of postsynaptic targets. For instance, climbing fibers are pruned from numerous branches to approximately seven (Sugihara, 2006; Fujita and Sugihara, 2013), and motor axon branches converge to a roughly consistent number depending on the type of muscle, forming so-called motor units (Brown et al., 1976; Tapia et al., 2012). Although this branch pruning is crucial for regulating the extent of divergence of information flow within neural circuits, the mechanisms of this regulation, particularly those determining the final number of postsynaptic targets innervated by each axon, remain incompletely understood.

To investigate the presynaptic mechanisms underlying circuit refinement during development, simple model systems that allow for facile assessment of complex axonal projection during development are invaluable. The chick ciliary ganglion (CG) synapse has long served as a valuable model for studying synaptic development (Carpenter, 1911; De Lorenzo, 1960; Yawo and Chuhma, 1993; Yawo and Momiyama, 1993; Dryer, 1994). Axons originating from the midbrain Edinger-Westphal nucleus project to ciliary neurons within the CG, forming large calyx-type presynaptic terminals. Synaptogenesis initiates around embryonic day 5 (E5), and by E8, all ciliary neurons receive inputs via multiple bouton-type presynaptic terminals. These multiple inputs are refined to a single calyx input by E14 (Landmesser and Pilar, 1972; Jacob, 1991), suggesting competition among axons innervating the same ciliary neuron. The advent of techniques such as *in ovo* electroporation for flexible gene expression (Odani et al., 2008; Egawa et al., 2013) and tissue clearing (Susaki et al., 2014; Katow et al., 2017; Egawa and Yawo, 2019) has enabled detailed analysis of axonal projections through single-axon tracing in this model circuit.

In this study, we investigated the developmental trajectory of axonal projections and the formation of calyx-type synapses within the chick CG. Single-axon tracing with Brainbow labeling and sparse expression strategies demonstrated that axons innervating the CG undergo extensive branch pruning by E14, converging to a one-to-one connection pattern with no branches and a single calyx. This refinement was disturbed by the presynaptic expression of genetically encoded silencing tools, suggesting that it is regulated in an activity-dependent manner. These features of circuit refinement are consistent with those observed in other model circuits, highlighting the potential of the CG as a simple and powerful model system for investigating presynaptic mechanisms of circuit refinement.

## Results

### Brainbow labeling revealed a homogenous one-to-one connection pattern of axons innervating the CG

We first investigated the number of postsynaptic targets innervated by individual preganglionic axons in the CG at E14, a stage at which calyx synapses are largely mature. To label preganglionic axons, we performed *in ovo* electroporation of a plasmid cocktail containing pCAGGS-Brainbow1.1-M and pCAG-Cre into the Edinger-Westphal nucleus at E2, resulting in stochastic expression of multiple fluorescent proteins in individual axons (Livet et al., 2007; Egawa et al., 2013). The dissected CGs were then tissue-cleared using CUBIC reagent-1 (Susaki et al., 2014) and imaged with confocal microscopy (Egawa and Yawo, 2019).

**Figure 1:**
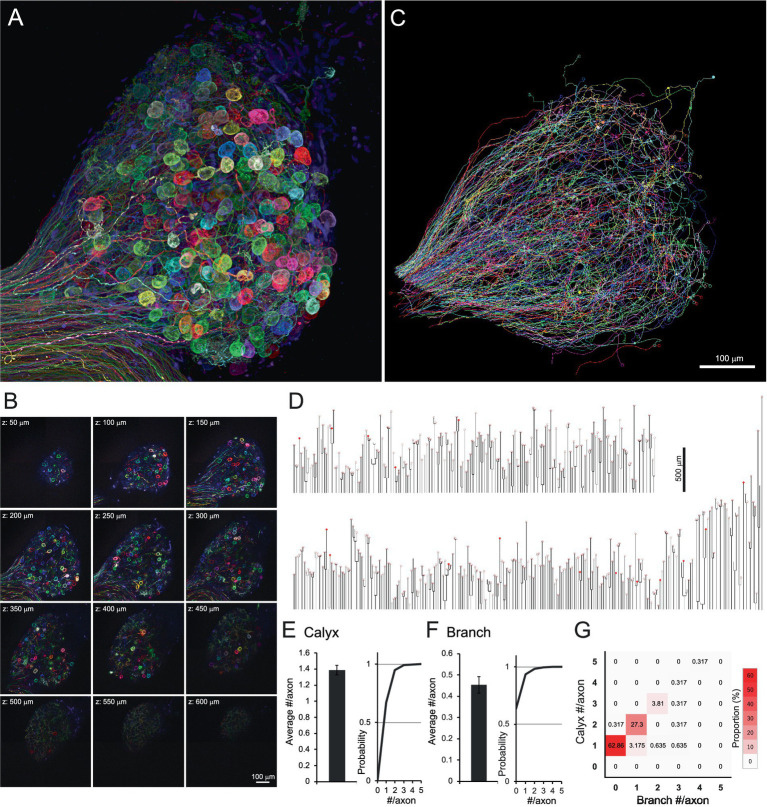
Brainbow labeling and tissue clearing visualized hundreds of axons innervating the chick CG. **(A,B)** Brainbow-labeled axons innervating a chick CG at E14. Confocal z-stack images were shown as a 3D reconstruction **(A)** and serial sectioning images at 50-μm intervals **(B)**. Numerous spherical structures are giant calyx-type presynaptic terminals enveloping their target ciliary neurons. **(C,D)** Single-axon tracing in CG. Each axon, randomly expressing multiple fluorescent proteins, was three-dimensionally traced using Neurolucida **(C)**. Traced axons were shown as dendrograms **(D)**. From one CG, 315 axons were traced, and 436 calyx-type presynaptic terminals were morphologically identified. On the dendrogram, open circles represent mature calyces (*n* = 420), and filled circles represent immature calyces (*n* = 16). **(E–G)** Morphometric analysis of traced axons. The number of calyx-type presynaptic terminals **(E)** and branches **(F)** per axon were shown with cumulative probability plots to illustrate the distribution of these data, and their frequency distribution is shown as a heatmap **(G)**. Warmer colors indicate higher frequency, with a single peak corresponding to axons exhibiting the one-to-one connection pattern with a single calyx and no branches (red box). Scale bars: 100 μm **(A–C)**; 500 μm **(D)**.

Figures 1A,B and Supplementary Videos 1–2 illustrate hundreds of axons entering a single CG, distinguishable by their distinct fluorescence signals. Of these, 315 axons projecting to ciliary neurons were traced using Neurolucida (Figures 1C and Supplementary Video 3). The traced axons were illustrated as dendrograms to visualize the overall morphology of individual axons, including branch points and calyx location (Figure 1D). The dendrograms revealed similarities in axonal morphology; the axon endings were almost always accompanied by calyx-type presynaptic terminals enveloping the soma of the ciliary neuron (filled and open circles on dendrograms). In addition, most axons had no or few branches. On average, axons had 1.38 ± 0.04 calyces (Figure 1E) and 0.45 ± 0.04 branches (Figure 1F). When the axons were classified according to the number of calyces and branches using a heatmap (Figure 1G), 62.86% of axons exhibited a one-to-one connection pattern, characterized by 1 calyx and 0 branches (red box in the second row from the bottom in the leftmost column).

### Single-axon tracing revealed developmental convergence of axonal projections via branch pruning and synapse maturation

We next asked how this one-to-one connection pattern is established during development. For single-axon tracing, the preganglionic axons were labeled densely with EGFP and sparsely with tdTomato by electroporating a plasmid cocktail containing pCAG-EGFP, pCAG-floxedSTOP-tdTomato, and a low concentration of pCAG-Cre. The CGs were dissected from E8 to E14 and imaged using two-photon microscopy.

**Figure 2:**
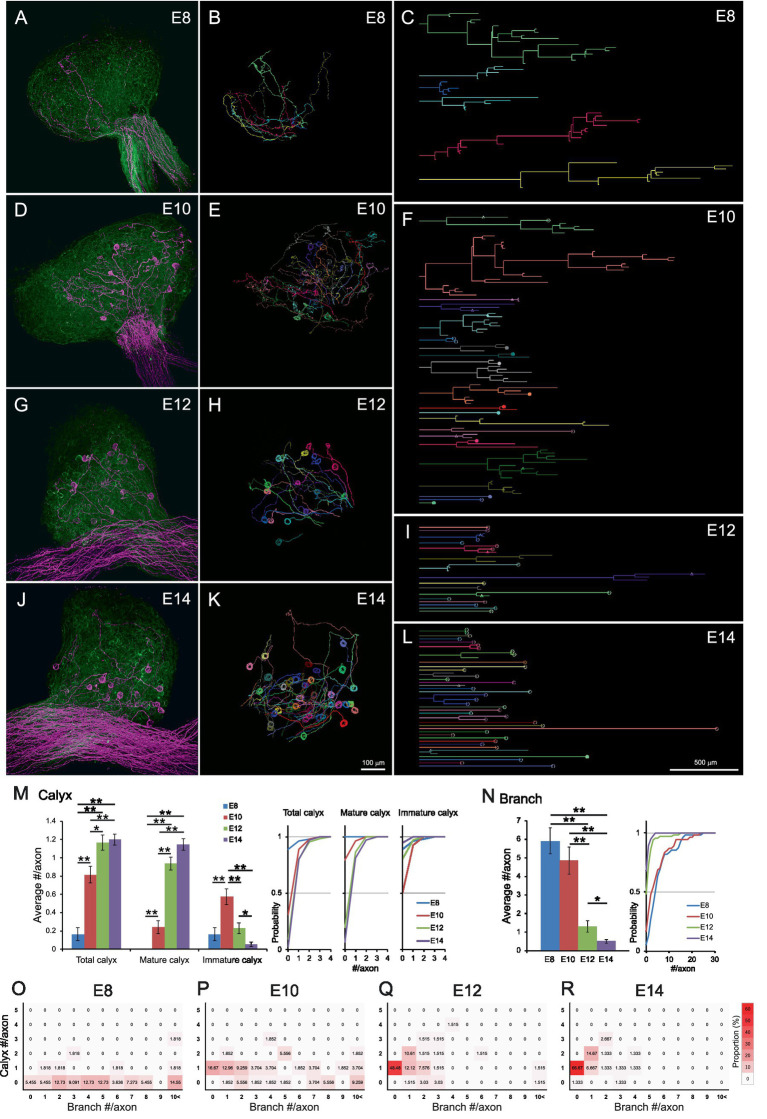
Axonal projections innervating CG converged to a one-to-one connection pattern during development. **(A–L)** Representative examples of single-axon tracing within developing CGs from E8 to E14. Axons innervating the CG were densely labeled with GFP (green) and sparsely labeled with tdTomato (magenta) and three-dimensionally reconstructed **(A,D,G,J)**. Each tdTomato-expressing axon was traced using Neurolucida **(B,E,H,K)**. The traced axons were shown as dendrograms **(C,F,I,L)**. Morphologically identified calyx-type presynaptic terminals were indicated on the dendrograms; open circles, mature calyces; filled circles, immature calyces; triangles, calyces with collateral. Scale bar: 100 μm **(A,B,D,E,G,H,J,K)**; 500 μm **(C,F,I,L)**. **(M,N)** Morphometric analysis of traced axons during E8–14. The number of calyx-type presynaptic terminals **(M)** and the number of branches **(N)** of each axon were shown with cumulative probability plots to illustrate the distribution of these data. Calyces were categorized by size: “mature” calyces cover more than half of the target neuron surface, whereas “immature” calyces partially cover it. **(O–R)** Heatmaps showing axon morphology. Traced axons at each stage (E8–E14) were sorted by the number of branches and calyx-type presynaptic terminals, and their frequency distributions were represented by color intensity. Warmer colors indicate higher frequency, showing a single peak corresponding to the one-to-one connection pattern (one calyx, no branches) emerges prominently from E12 onward. Statistical analysis: Kruskal-Wallis test with Steel-Dwass *post hoc* test: *p < 0.05, **p < 0.005. Sample sizes: E8: *n* = 9 calyces, 55 axons, 8 CGs; E10: *n* = 44 calyces, 54 axons, 5 CGs; E12: *n* = 77 calyces, 66 axons, 6 CGs; E14: *n* = 90 calyces, 75 axons, 4 CGs.

At E8, axons exhibited numerous branches and few morphologically identifiable calyx-type presynaptic terminals (Figures 2A–C and Supplementary Video 4). Given that all ciliary neurons at this stage receive input via multiple small bouton-type synapses on short dendrites, (Landmesser and Pilar, 1972; Dryer, 1994), each branch likely possesses multiple small presynaptic terminals. By E10, calyx-type presynaptic terminals began to appear (Figures 2D–F and Supplementary Video 5), but they were often immature, partially covering the postsynaptic cell. At E12, the proportion of mature calyces, which cover more than half of the postsynaptic cell surface area, increased, concomitant with a reduction in branching (Figures 2G–I and Supplementary Video 6). This trend progressed further at E14 (Figures 2J–L and Supplementary Video 7). Quantitative analysis of axonal morphology revealed that the number of calyces per axon significantly increased between E8 and E12 (E8: 0.16 ± 0.07, E10: 0.82 ± 0.09, E12: 1.17 ± 0.09, E14: 1.20 ± 0.06, *p* = 2.8 × 10^−8^ for E8 vs. E10, *p* = 0.021 for E10 vs. E12) (Figure 2M). Among them, mature calyces increased prominently between E10 and E12 (E8: 0, E10: 0.24 ± 0.07, E12: 0.94 ± 0.07, E14: 1.15 ± 0.06, *p* = 0.0025 for E8 vs. E10, *p* = 2.7 × 10^−9^ for E10 vs. E12), while the number of immature calyces peaked at E10 and subsequently decreased with development (E8: 0.16 ± 0.07, E10: 0.57 ± 0.09, E12: 0.23 ± 0.06, E14: 0.05 ± 0.03, *p* = 0.00013 for E8 vs. E10, *p* = 0.0032 for E10 vs. E12, *p* = 0.042 for E12 vs. E14). The number of branches also decreased substantially between E10 and E12 (E8: 5.91 ± 0.70, E10: 4.85 ± 0.74, E12: 1.30 ± 0.31, E14: 0.51 ± 0.11, *p* = 3.7 × 10^−6^ for E10 vs. E12, *p* = 0.042 for E12 vs. E14) (Figure 2N). Heatmaps classifying axon morphology on the basis of the numbers of calyces and branches revealed a distinct peak of one-to-one connection pattern (1 calyx, 0 branches) after E10 (E8: 0%, E10: 16.67%, E12: 48.48%, E14: 66.67%) (Figures 2O–R), demonstrating developmental convergence through calyx synapse maturation and branch pruning.

**Figure 3:**
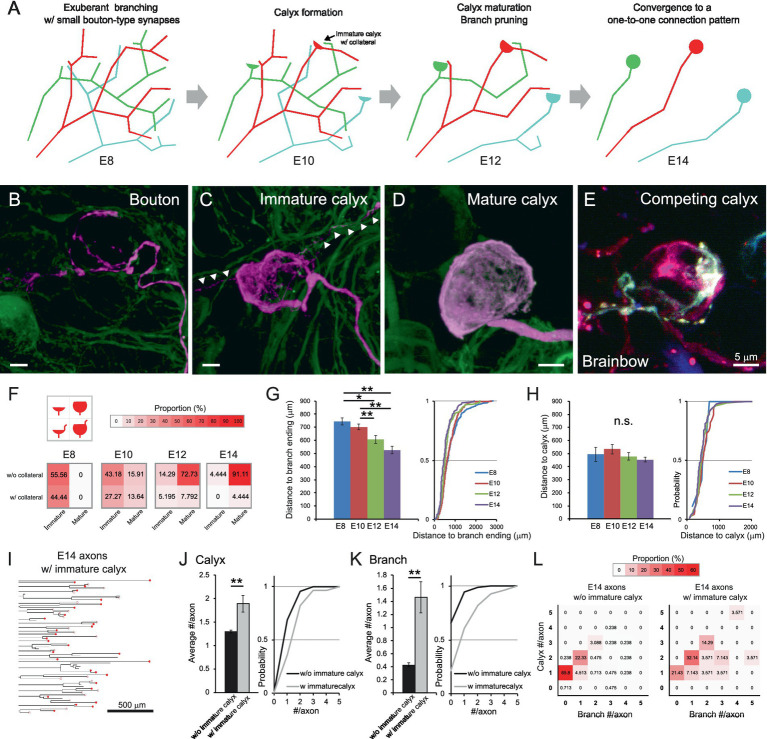
Maturation of calyx-type presynaptic terminals via developmental pruning of axon arborization. **(A)** Schematic diagram of the developmental refinement of axonal projections innervating the CG. At E8, the axon has abundant branches with bouton-type synapses, and postsynaptic targets receive multiple inputs from these axons. At E10, one of the synapses on the axon begins to specialize into a calyx, of which around 40% have collaterals. From E12, the calyx maturation and branch pruning progress, and at E14, they converge into a one-to-one connection pattern. **(B–E)** Representative images of presynaptic terminals in developing CGs. Examples include a putative bouton-like ending about to specialize in calyx. **(B)** and an immature calyx with short collaterals **(C)** at E10, and a mature calyx **(D)** at E14. Arrowheads indicate collaterals associated with immature calyces. GFP is shown in green, and tdTomato in magenta. Competing immature calyces (red and cyan) on the same target neuron were detected in an E10 CG labeled with Brainbow **(E)**. Scale bar: 5 μm. **(F)** Heatmaps showing the proportions of four calyx types from E8 to E14. Calyces were categorized by size and the presence of collaterals, and their proportions were represented by color intensity. Warmer colors indicate higher frequency, showing that calyces progressively converge toward a typical mature morphology lacking collaterals and covering more than half of the postsynaptic cell surface. **(G,H)** Relationship between the axon length and the calyx positions during development. The distance along the axon from the where the oculomotor nerve enters the CG to the distal ends of axonal branches **(G)** and to each calyx **(H)** were shown with cumulative probability plots to illustrate the distribution of these data. **(I–L)** Morphometric analysis focusing on axons with immature calyces at E14. The dendrograms showed 28 axons with immature calyces extracted from the 449 axons traced at E14 in this study **(I)**. The number of calyx-type presynaptic terminals **(J)** and branches **(K)** per axon were compared between axons with and without immature calyces and shown with cumulative probability plot to illustrate the distribution of these data. The frequency distribution was shown in the heatmaps as color intensity **(L)**. Warmer colors indicate higher frequency, showing that in axons with immature calyces, the peak has shifted to the box corresponding to a pattern of two calyces and one branch. Sample sizes **(I–L)**: Axons without immature calyces at E14: *n* = 547 calyces, 421 axons, 13 CGs; Axons with immature calyces at E14: *n* = 53 calyces, 28 axons, 13 CGs. Statistical analysis: Kruskal-Wallis test with Steel-Dwass post hoc test **(F,G)** and Wilcoxon rank sum test **(I,J)**: *p < 0.05, **p < 0.005, n.s., not significant. Sample sizes **(E–G)**: E8: *n* = 9 calyces, 55 axons, 379 endings, 8 CGs; E10: *n* = 44 calyces, 54 axons, 318 endings, 5 CGs; E12: *n* = 77 calyces, 66 axons, 154 endings, 6 CGs; E14: *n* = 90 calyces, 75 axons, 119 endings, 4 CGs.

Further analysis provided insights into the underlying rules of the developmental refinement of axonal projections innervating the CG (Figure 3A). As maturation progressed, presynaptic terminals increased in size and expanded their coverage over the postsynaptic soma (Figures 3B–D and Supplementary Videos 8–10). In addition, collaterals associated with the calyces (Figures 3C arrowheads) became less prominent. Brainbow labeling visualized two differently colored immature calyces formed on the same postsynaptic cell at E10 (Figure 3E and Supplementary Video 11), providing evidence for competition among presynaptic terminals. To quantitatively assess calyx maturation, we classified calyces into four subtypes based on their maturity and the presence or absence of collaterals, visualizing their frequency at each developmental stage using heatmap (Figure 3F). At E8 and E10, approximately 40% of calyces exhibited collaterals (bottom two boxes), whereas by E14, the majority of these had disappeared, and nearly 90% of calyces exhibited the morphology of a typical mature form (top-right box). Similar transient collaterals are also reported in the calyx of Held, representing the features of early calyces (Rodríguez-Contreras et al., 2008). These collaterals may be remnants of distal segments of the presynaptic terminal formed in the middle of axon at younger stages. Consistent with this interpretation, the distance along the axon from the entry point of oculomotor nerve in the CG to the distal ends of axonal branches gradually decreased during development (E8: 744.2 ± 26.4 μm, E10: 702.3 ± 21.4 μm, E12: 606.6 ± 31.5 μm, E14: 525.4 ± 29.2 μm, *p* = 0.0033 for E10 vs. E12) (Figure 3G), whereas the distance to the calyx location remained relatively constant (E8: 495.1 ± 54.0 μm, E10: 533.5 ± 37.2 μm, E12: 478.2 ± 29.5 μm, E14: 452.9 ± 19.0 μm, *p* > 0.14) (Figure 3H). Notably, the small population of axons retaining immature calyces at E14 (axons with immature calyx: 28/449 axons) exhibited distinct morphological features (Figure 3I). When they were compared with the remaining group (axons without immature calyx: 421/449 axons), they showed significantly higher numbers of both calyces (1.3 ± 0.03 and 1.89 ± 0.18 for axons without and with immature calyx, respectively, *p* = 1.9 × 10^−8^) (Figure 3J) and branches per axon (0.43 ± 0.03 and 1.46 ± 0.24 for axons without and with immature calyx, respectively, *p* = 1.0 × 10^−^⁵) (Figure 3K). Consistent with these findings, heatmap-based classification of axonal morphology revealed that the most frequent pattern of axons with immature calyx consisted of 2 calyces and 1 branch (32.14%) (Figure 3L), suggesting that retention of immature calyces confers an advantage in maintaining connections to multiple targets.

### Presynaptic silencing disrupted the refinement of axonal projections innervating the CG

**Figure 4:**
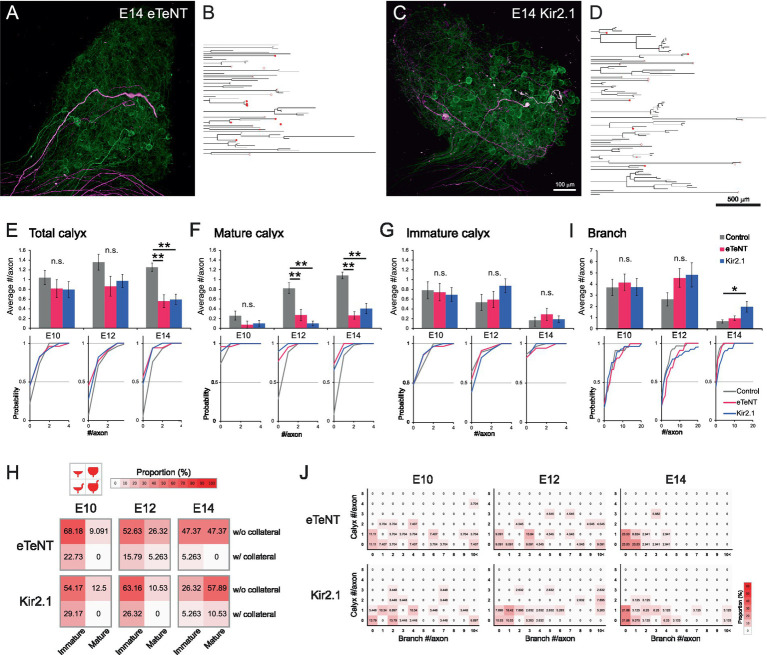
Presynaptic silencing prevented maturation of calyx-type presynaptic terminals. **(A–D)** Representative examples of axonal projections with the expression of genetically encoded tools for silencing neural activity at E14. These silenced axons displayed a noticeable reduction in a typical projection pattern that exhibits an unbranched morphology with a single mature calyx terminal. eTeNT **(A)** or Kir2.1 **(C)** was sparsely co-expressed with tdTomato (magenta). Axons not expressing these tools were densely labeled with GFP (green). The traced axons expressing eTeNT **(B)** or Kir2.1 **(D)** were shown as dendrograms. Morphologically identified calyx-type presynaptic terminals were indicated on the dendrograms: open circles, mature calyces; filled circles, immature calyces. Scale bar: 100 μm **(A,C)**; 500 μm **(B,D)**. See also Figures 2J,L for visual reference. **(E–G)** Analysis of the number of calyces per axon from E10 to E14. Axons expressing eTeNT or Kir2.1 were compared with the control group. The numbers of all types of calyces **(E)**, mature calyces **(F)** and immature calyces **(G)** per axon during development were shown with cumulative probability plots to illustrate the distribution of these data. Gray: control; Red: eTeNT; Blue: Kir2.1. **(H)** Heatmaps showing the proportions of four calyx types. Calyces at each stage were categorized by size and the presence of collaterals, and their proportions are represented by color intensity. Warmer colors indicate higher frequency, showing that convergence to typical mature calyces was impeded in silenced axons. See also Figure 3F for visual reference. **(I)** Analysis of the number of branches per axon from E10 to E14. Axons expressing eTeNT or Kir2.1 were compared to control group and are shown with cumulative probability plots to illustrate the distribution of these data. Gray: control; Red: eTeNT; Blue: Kir2.1. **(J)** Heatmaps showing axonal morphology. Traced axons at each stage were sorted by the number of branches and calyx-type presynaptic terminals, and their frequency distribution was represented by color intensity. Warmer colors indicate higher frequency, showing that developmental convergence to one-to-one connections is impeded in silenced axons. See also Figures 2P–R for visual reference. Statistical analysis: Kruskal-Wallis test with Steel-Dwass *post hoc* test: *p < 0.05, **p < 0.005, n.s., not significant. Sample sizes: E10 control: *n* = 23 axons, 24 calyces, 4 CGs; E12 control: *n* = 28 axons, 38 calyces, 5 CGs; E14 control: *n* = 59 axons, 74 calyces, 7 CGs; E10 eTeNT: *n* = 27 axons, 22 calyces, 6 CGs; E12 eTeNT: *n* = 22 axons, 19 calyces, 4 CGs; E14 eTeNT: *n* = 34 axons, 19 calyces, 6 CGs; E10 Kir2.1: *n* = 29 axons, 23 calyces, 3 CGs; E12 Kir2.1: *n* = 39 axons, 38 calyces, 3 CGs; E14 Kir2.1: *n* = 32 axons, 19 calyces, 9 CGs.

Activity-dependent competition may drive the maturation from multiple small bouton-type synapses to a single calyx-type synapse in the CG. To investigate the role of neuronal activity in the refinement of axonal projections, we used two genetically encoded silencing tools (Wiegert et al., 2017): enhanced tetanus neurotoxin light chain (eTeNT; Kinoshita et al., 2012), which inhibits vesicular release, and Kir2.1 (Ishii et al., 1994; Mizuno et al., 2007), an inward rectifier potassium channel which hyperpolarizes the membrane potential. We sparsely co-expressed tdTomato with either eTeNT or Kir2.1 in preganglionic axons by electroporating a plasmid cocktail containing pCAG-floxedSTOP-tdTomato-P2A-eTeNT or pCAG-floxedSTOP-tdTomato-P2A-Kir2.1, along with pCAG-EGFP and a low concentration of pCAG-Cre. The CGs were dissected from E10–14, and the morphology of individual axons was assessed (Figures 4A–D and Supplementary Videos 12–13).

The expression of eTeNT or Kir2.1 did not affect axonal projection patterns at E10. However, from E12 onwards, maturation of calyces was impaired, resulting in a reduced number of total and mature calyces per axon at E14; in control, eTeNT and Kir2.1, respectively, the numbers of calyces were 1.25 ± 0.09, 0.56 ± 0.13 and 0.59 ± 0.11 for total (*p* = 7.7 × 10^−7^ for control vs. eTeNT, *p* = 1.1 × 10^−^⁵ for control vs. Kir2.1) (Figure 4E); 1.08 ± 0.07, 0.26 ± 0.08 and 0.41 ± 0.11 for mature (*p* = 7.6 × 10^−8^ for control vs. eTeNT, *p* = 4.4 × 10^−^⁵ for control vs. Kir2.1) (Figure 4F); 0.17 ± 0.06, 0.29 ± 0.13 and 0.19 ± 0.07 for immature (*p* > 0.89) (Figure 4G). Consistent with these findings, heatmaps classifying calyx subtypes showed that the proportion of typical mature calyces at E14 was also reduced in silenced axons (top-right box; control: 85.14%, eTeNT: 47.37%, Kir2.1: 57.89%) (Figure 4H). Furthermore, the number of branches increased specifically in Kir2.1-expressing axons at E14 (control: 0.66 ± 0.14, eTeNT: 0.94 ± 0.20, Kir2.1: 1.97 ± 0.48, *p* = 0.046 for control vs. Kir2.1) (Figure 4I). Consequently, heatmap-based classification of axonal morphology revealed that the expression of either eTeNT or Kir2.1 impaired the developmental convergence of axonal morphology to a one-to-one connection pattern (1 calyx, 0 branches) (control: 59.32%, eTeNT: 23.53%, Kir2.1: 21.88%) (Figure 4J). These results indicate that presynaptic activity plays a crucial role in the refinement of axonal projections.

## Discussion

In this study, we elucidated the developmental refinement of preganglionic axonal projection patterns in the chick CG, a long-established model system for synapse research. Using single-axon tracing with Brainbow labeling and sparse expression strategy, we demonstrated that preganglionic axons undergo extensive branch pruning between E8 and E14, which coincides with the switching from multiple inputs via bouton synapses to a single input via a calyx synapse, ultimately converging to a one-to-one connection pattern. Furthermore, by using genetically encoded silencing tools, we demonstrated that the refinement of axonal projections innervating CG is driven in an activity-dependent manner.

### Developmental convergence of axonal projection patterns in the CG

Consistent with other model circuits exhibiting activity-dependent synaptic competition, such as the neuromuscular junction and the cerebellar climbing fiber-Purkinje cell synapse, axonal branches in the CG underwent extensive developmental pruning, converging on a small subset of postsynaptic targets for each axon. This convergence of axonal projection patterns indicates that “winner” axons, defined as axons successfully maintaining synaptic contact with postsynaptic targets, do not monopolize a large number of targets, suggesting that mechanisms other than synaptic competition may be involved in this process. Such mechanisms could be related to limitations in synaptic resources (Barber and Lichtman, 1999). This idea is supported by a previous work at the neuromuscular junction demonstrating that axons with fewer branches have an advantage in synaptic competition (Kasthuri and Lichtman, 2003) and also by the tendency observed in this study for axons with immature calyces to possess more calyces and branches. Specifically, the maturation of a large calyx enveloping the postsynaptic cell likely requires a greater allocation of such resources, which may underlie the drastic convergence of projection patterns to a single calyx per axon. The precise nature of these resources warrants further investigation. For example, mitochondria may represent one potential component of these synaptic resources; they are abundant within calyx-type presynaptic terminals and are known to modulate synaptic transmission efficiency (De Lorenzo, 1960; Landmesser and Pilar, 1972; Thomas et al., 2019). Elucidating the composition of these resources may pave the way for future manipulations of the number of target cells innervated by axons.

### Activity-dependent refinement of axonal innervation to the CG

The maturation of calyces was impaired in the preganglionic axons sparsely expressing either eTeNT or Kir2.1, genetically encoded tools for silencing neuronal activity (Wiegert et al., 2017). This result suggests that these silenced axons were outcompeted by other axons innervating the same postsynaptic target, consistent with the concept of activity-dependent synaptic competition. On the other hand, the number of immature calyces per axon remained largely unaffected by either eTeNT or Kir2.1 expression, suggesting that initial formation of calyces can proceed independently of presynaptic activity. Interestingly, a small subset of axons still formed mature calyces despite expressing the silencing tools, which might occur through pruning of competitor axons by the mechanisms other than synaptic competition, such as those mediated by glial cells (Park and Chung, 2023) or by the axon itself to allocate resources for calyx maturation to other branches (Brill et al., 2016).

The effects of eTeNT and Kir2.1 expression on axonal projection patterns at E14 differed; Kir2.1 expression resulted in a significantly greater number of remaining branches. This could be attributed to the fact that calcium influx is required for axonal retraction (Wang et al., 2012), while eTeNT does not directly prevent action potential generation in the axons. These may indicate that action potential is an important factor not only for synaptic competition but also for promoting the retraction of losing axons.

### Technical limitations of single-axon tracing

A technical limitation of single-axon tracing was the difficulty in morphologically identifying bouton-type presynaptic terminals along axons, unlike the readily distinguishable calyces with their characteristic sheet-like coverage of the postsynaptic neuron. Therefore, we could neither assess the number of postsynaptic targets innervated by individual axons at E8 nor determine whether branches lacking calyces at E14 were undergoing retraction. Furthermore, the traceability of individual axons depends on the density and complexity of labeled axons; under our current imaging conditions, tracing of CGs with Brainbow labeling at younger stages is extremely challenging. In this study, we minimized spherical aberration by employing CUBIC reagent-1, which is used for delipidation in older version of CUBIC method (Susaki et al., 2014), chosen specifically to reduce refractive index mismatch with our available long-working-distance, high-numerical-aperture silicone-immersion and water-immersion objectives. However, some refractive index mismatch inevitably remained, causing a loss of resolution as the imaging depth increased. To address these technical challenges, a new approach that combines expansion microscopy with immunohistochemical labeling may prove beneficial (Park et al., 2018; Gao et al., 2019). This approach could enable more accurate axon segmentation and identification of presynaptic active zones, potentially facilitating automated, high-throughput single-axon tracing (Liu et al., 2022; Cai et al., 2024).

## Conclusion

In summary, this study demonstrates that the chick CG shares fundamental characteristics of circuit remodeling with other model circuits, in terms of the convergence of axonal projections onto a limited number of postsynaptic targets and activity-dependent synaptic competition. Given its structural simplicity and flexibility of genetic manipulation, the chick CG holds promise as a valuable model system for investigating presynaptic mechanisms underlying circuit remodeling.

## Materials and methods

### Chick embryo and tissue preparation

Fertilized White Leghorn chicken eggs were incubated at 38°C in a humidified incubator until reaching the desired developmental stages [Hamburger and Hamilton (HH) stages; Hamburger and Hamilton (1951)]. At each stage (E8: HH stage 33–34; E10: HH stage 36; E12: HH stage 38; E14: HH stage 40), embryos were decapitated, and the bilateral CGs with attached oculomotor nerves were dissected and fixed in 4% paraformaldehyde in PBS for 1.5 h. Fixed CGs were tissue-cleared for 1 day prior to imaging using CUBIC reagent-1 (Susaki et al., 2014) containing 25% (w/w) urea (35904–45, Nacalai Tesque), 25% (w/w) N,N,N′,N′-tetrakis(2-hydroxypropyl) ethylenediamine (T0781, Tokyo Chemical Industry), and 15% (w/w) polyethylene glycol mono-p-isooctylphenyl ether/Triton X-100 (25987–85, Nacalai Tesque).

### Plasmids

Previously described plasmids included pCAG-Brainbow1.1 M, pCAG-EGFP, pCAG-floxedSTOP-tdTomato, and pCAG-Cre (Egawa and Yawo, 2019). pCAG-floxedSTOP-tdTomato-P2A-eTeNT and pCAG-floxedSTOP-tdTomato-P2A-Kir2.1 were constructed by inserting the following sequences into the pCAG-floxedSTOP-tdTomato backbone using In-Fusion® Snap Assembly Master Mix (Takara Bio): eTeNT (gift from Kazuto Kobayashi; Kinoshita et al., 2012) and rabbit Kir2.1 (NM_001082198; gift from Kuniaki Ishii; Ishii et al., 1994). All constructs were verified by Sanger sequencing.

### *In ovo* electroporation

Plasmid DNA was introduced into chick embryos as described previously (Egawa and Yawo, 2019). Briefly, plasmid cocktails were co-injected with Fast Green into the midbrain ventricular space of E2 chick embryos (HH stage 14–16). The concentration of plasmids was generally 0.2–0.3 μg/μL, except for sparse expression conditions, in which pCAG-Cre was diluted to 0.001 μg/μL. Electroporation was performed using a pair of parallel electrodes placed on the embryonic brain to target the midbrain region. Four rectangular pulses (25 V, 50 ms duration, 950 ms interval) were delivered using a CUY21EDIT electroporator (BEX). Morphological abnormalities were not observed in the cell bodies of electroporated neurons for either construct (data not shown). For experiments requiring sparse expression, CGs with tdTomato-labeled axons at appropriate sparsity were identified under a fluorescence stereomicroscope (Olympus MVX10) and selected for subsequent imaging.

### Image acquisition and analysis

Tissue-cleared CGs were embedded as previously reported (Egawa and Yawo, 2019) and imaged using confocal or two-photon microscopy: Olympus FV3000 with a 30× silicone-immersion objective (UPLSAPO30XS) for Figure 1, Nikon A1R-MP+ with a 16× water-immersion objective (CFI75 LWD 16 × W) for Figures 2, 4, Zeiss LSM710 with a 63× oil-immersion objective (Plan-Apochromat 63x/1.4 Oil) for Figure 3. Z-stack images were converted to 16-bit TIFF format using ImageJ (http://rsb.info.nih.gov/ij/) and reconstructed in three dimensions with FluoRender software (Wan et al., 2017). Individual axons were manually traced using Neurolucida software (MBF Bioscience). Those that could not be traced with sufficient accuracy were not considered for analysis; these included thin axons with highly intricate arborization projecting to the small cell population of choroid neurons in the ventral region of the CG (Marwitt et al., 1971) (Supplementary Video 14). The traced axons were quantitatively analyzed using Neurolucida Explorer software (MBF Bioscience) to generate dendrograms that summarize the morphology of each axon and to extract parameters such as number of branch points, length along axons, and associated calyces. The resulting data were further processed using Excel (Microsoft) to generate bar graphs, cumulative probability plots, and color-intensity heatmaps.

### Statistics

Statistical analysis was performed using R software (http://cran.r-project.org/). Data normality was assessed with the Shapiro–Wilk test. As most data exhibited non-normal distributions, non-parametric tests were used: Wilcoxon rank sum test for two-group comparisons, Kruskal-Wallis test for comparisons between two or more groups, and Steel-Dwass *post hoc* test for pairwise comparisons. Statistical significance was set at *p* < 0.05. Data are presented as mean ± standard error of the mean (SEM).

## Data Availability

The raw data supporting the conclusions of this article will be made available by the authors, without undue reservation.
